# Distinctive Evasion Mechanisms to Allow Persistence of *Borrelia burgdorferi* in Different Human Cell Lines

**DOI:** 10.3389/fmicb.2021.711291

**Published:** 2021-10-12

**Authors:** Kati Karvonen, Jonna Nykky, Varpu Marjomäki, Leona Gilbert

**Affiliations:** ^1^Department of Biological and Environmental Science, Nanoscience Center, University of Jyväskylä, Jyväskylä, Finland; ^2^Te?ted Oy Ltd., Jyväskylä, Finland

**Keywords:** lyme borreliosis, pleomorphic forms, immune evasion, microscopy, persist

## Abstract

Lyme borreliosis is a multisystemic disease caused by the pleomorphic bacteria of the *Borrelia burgdorferi sensu lato* complex. The exact mechanisms for the infection to progress into a prolonged sequelae of the disease are currently unknown, although immune evasion and persistence of the bacteria in the host are thought to be major contributors. The current study investigated *B. burgdorferi* infection processes in two human cell lines, both non-immune and non-phagocytic, to further understand the mechanisms of infection of this bacterium. By utilizing light, confocal, helium ion, and transmission electron microscopy, borrelial infection of chondrosarcoma (SW1353) and dermal fibroblast (BJ) cells were examined from an early 30-min time point to a late 9-days post-infection. Host cell invasion, viability of both the host and *B. burgdorferi*, as well as, co-localization with lysosomes and the presence of different borrelial pleomorphic forms were analyzed. The results demonstrated differences of infection between the cell lines starting from early entry as *B. burgdorferi* invaded BJ cells in coiled forms with less pronounced host cell extensions, whereas in SW1353 cells, micropodial interactions with spirochetes were always seen. Moreover, infection of BJ cells increased in a dose dependent manner throughout the examined 9 days, while the percentage of infection, although dose dependent, decreased in SW1353 cells after reaching a peak at 48 h. Furthermore, blebs, round body and damaged *B. burgdorferi* forms, were mostly observed from the infected SW1353 cells, while spirochetes dominated in BJ cells. Both infected host cell lines grew and remained viable after 9 day post-infection. Although damaged forms were noticed in both cell lines, co-localization with lysosomes was low in both cell lines, especially in BJ cells. The invasion of non-phagocytic cells and the lack of cytopathic effects onto the host cells by *B. burgdorferi* indicated one mechanism of immune evasion for the bacteria. The differences in attachment, pleomorphic form expressions, and the lack of lysosomal involvement between the infected host cells likely explain the ability of a bacterium to adapt to different environments, as well as, a strategy for persistence inside a host.

## Introduction

*Borrelia burgdorferi*, the causative agent of Lyme borreliosis (LB), is a pleomorphic bacterium transmitted *via Ixodes* ticks (Burgdorfer et al., 1982). *Borrelia* bacteria can be found globally and the main species causing LB are *B. burgdorferi sensu stricto*, *Borrelia garinii*, and *Borrelia afzelii*. In normal culture conditions, *Borrelia* is found in the parental spirochete form with a length varying from 10 to 30 μm, as well as, forming vesicles or blebs, as a metabolically inactive round body (RB) and even in biofilms (Meriläinen et al., 2015). Additionally, these pleomorphic forms can be induced by unfavorable culture conditions such as changes in pH, osmotic pressure, temperature, and even with antibiotic treatment (Kersten et al., 1995; Murgia and Cinco, 2004; Srivastava and De Silva, 2009; Meriläinen et al., 2015; Sharma et al., 2015). However, upon return to optimal culture conditions *Borrelia* can revert back to spirochetes (Brorson and Brorson, 1998; Gruntar et al., 2001; Meriläinen et al., 2015; Sharma et al., 2015).

Lyme borreliosis is the most common vector-borne disease in North America and Europe (Mead, 2015). The infection can lead to a multisystemic disorder with signs and symptoms ranging from mild flu-like symptoms and erythema migrans rash, to autoimmune-like disorders such as acrodermatitis chronica atrophicans, Lyme arthritis, and neurological, or cardiac impairments (Wormser et al., 2006; Sehgal and Khurana, 2015). The variety of signs and symptoms are due both to bacterial dissemination into distal tissues, as well as, local inflammatory reactions of the immune system to the bacterial proteins (reviewed in Hyde, 2017; Radolf et al., 2020). Antibiotics usually eradicate *Borrelia* if treatment is given early on in the infection. However, patients who suffer from what is termed post-treatment Lyme disease syndrome (Stanek et al., 2012) or chronic Lyme (Shor et al., 2019) remain. These patients have previously received antibiotic treatment but continue to exhibit different LB related clinical symptoms. *Borrelia* persistence in humans is currently under debate, although the pleomorphic forms of *Borrelia* are thought to play a major role in the persistence of the bacteria (Miklossy et al., 2008; Sharma et al., 2015; Rudenko et al., 2019). However, the exact mechanisms resulting in the long-term sequelae of the disease are currently undetermined.

Pathogens have several mechanisms for entering non-phagocytic cells. Smaller pathogens fit inside, for instance, caveolae or clathrin-coated vesicles, while larger ones internalize *via* more sizeable cellular compartments and progress through such endocytosis pathways as macropinocytosis (Cossart and Helenius, 2014). *Borrelia* have been demonstrated to invade a variety of human cells, for instance, endothelial cells (Comstock and Thomas, 1991; Ma et al., 1991), dermal fibroblasts (Klempner et al., 1993; Rozwadowska et al., 2017), synovial cells (Girschick et al., 1996), and neural cells (Livengood and Gilmore, 2006; Miklossy et al., 2008), among others. Furthermore, it has been demonstrated that *Borrelia* can be internalized by human cells without affecting cell viability, and this has been thought to play a role in host immune evasion (Ma et al., 1991; Girschick et al., 1996; Livengood and Gilmore, 2006). The ability to evade the host immune system is an integral part of *Borrelia* survival and dissemination throughout the body. How exactly *Borrelia* escapes being noticed by the immune system is yet to be resolved. Nevertheless, there are known *Borrelia* immune escape strategies including using tick proteins as a disguise, active suppression of host immune system, antigenic variation of the bacterial membrane proteins and altering its shape into a different pleomorphic form, among others (reviewed in Embers et al., 2004; Berndtson, 2013; Rudenko et al., 2019). Further understanding of the infection mechanisms of these bacteria by investigating the cellular invasion and its outcome in non-phagocytic cells is crucial. In this study, *Borrelia* infection of two non-immune and non-phagocytic human cell lines was examined. The results illustrated that *B. burgdorferi* could maintain a variety of intracellular locations inside non-phagocytic human cells during extended infection periods without instigating cytopathic effects. Moreover, differences in *B. burgdorferi* invasion processes, as well as, in infectivity between the two human cell lines were apparent. Furthermore, different borrelial pleomorphic forms were visualized inside the infected human cells. Consequently, though *B. burgdorferi* infected these human cells in a divergent manner, both the invasion and persistence with pleomorphic forms inside non-phagocytic host cells outlined strategies for *Borrelia* to avoid clearance by the immune system, and thus, initiate a persisting infection.

## Materials and Methods

### Human Cell Cultures

Normal dermal fibroblast cell line (BJ, CRL-2522) and chondrosarcoma cell line (SW1353, HTB-94) were purchased from American Type Culture Collection. Dermal fibroblasts have been previously used in investigations of borrelial infection (Georgilis et al., 1992). Furthermore, the use of chondrosarcoma cells in osteoarthritis research is well documented (Chen et al., 2017; Liu et al., 2017). Therefore, these cell lines, BJ and SW1353, were utilized for their relevance as a disease-related model for skin manifestations and arthritis, respectively. The SW1353 cells were grown at +37°C with 100% air in Leibovitz’s L-15 media (Sigma), while the BJ cells were at +37°C, 5% CO_2_ in Eagle’s minimum essential media (Sigma) as instructed by the manufacturers. Both media were supplemented with 10% fetal bovine serum (Gibco), 2 mM L-glutamine (Gibco), and 100 IU/ml penicillin/0.2 mg/ml streptomycin (Gibco) antibiotic cocktail. Sodium pyruvate (1 mM, Gibco) was also added to the BJ media.

### Bacteria Cultures

The infectious *B. burgdorferi* strain GCB726 with green fluorescent protein (GFP) (hereafter referred to as *B. burgdorferi*), a generous gift from George Chakonas, University of Calgary, Canada (Moriarty et al., 2008), was grown, and round bodies (RBs) were induced as previously described (Meriläinen et al., 2015). In all experiments, low-passage number cells (≤p8) in log phase were utilized.

### Infection Protocol

In the proceeding experiments (“Helium Ion Microscopy,” “*Borrelia burgdorferi* Infection Assay and Wheat Germ Agglutinin Staining,” “Human Cell Viability Assays,” “*Borrelial* Survival,” “Transmission Electron Microscopy,” and “Co-localization and Green Fluorescent Protein Signal Analyses With Immunolabeling Procedure” sections) the cell density, multiplicity of infections (MOIs) and time of infection are specific for each experiment. In general, human cells were seeded in antibiotic-free media a day before the infection in order for the cells to fully attach to the culture dishes. *B. burgdorferi* was counted using a C-Chip DHC-N01 Disposable Hemocytometer (System Neubauer Improved; Digital Bio). The specific MOIs (see “Helium Ion Microscopy,” “*Borrelia burgdorferi* Infection Assay and Wheat Germ Agglutinin Staining,” “Human Cell Viability Assays,” “*Borrelial* Survival,” “Transmission Electron Microscopy,” and “Co-localization and Green Fluorescent Protein Signal Analyses With Immunolabeling Procedure” sections) of *B. burgdorferi* were centrifuged 1,000 × *g* for 15 min and resuspended into the antibiotic-free media of each cell line. Before adding *Borrelia*, the human cells were washed once with +37°C PBS. *B. burgdorferi* entry into the human cells was synchronized with 1-h incubation on ice. After ice incubation, antibiotic-free media was added according to standard culture plates used, and the infection was allowed to proceed according to each respective experiment (sections “Helium Ion Microscopy,” “*Borrelia burgdorferi* Infection Assay and Wheat Germ Agglutinin Staining,” “Human Cell Viability Assays,” “*Borrelial* Survival,” “Transmission Electron Microscopy,” and “Co-localization and Green Fluorescent Protein Signal Analyses With Immunolabeling Procedure”).

### Helium Ion Microscopy

Helium ion microscopy (HIM) (Zeiss Orion Nanofab) was utilized to visualize early human cell attachment and invasion by *B. burgdorferi*. The 100,000 cells were seeded onto coverslips with integrated grids in them (High Precision microscope cover glasses, 28 mm, No. 1.5H, Paul Marienfeld GmbH & Co., Germany) in six-well culture plates and incubated overnight accordingly. As controls, samples with only human cells, spirochetes and RBs, individually, were used. The gridded areas of the cover slips used for bacterial controls were coated with 50-μl drop of poly-L-lysine (P8920, Sigma) according to the instructions of the manufacturer (Sigma). Borrelial control samples were allowed to attach onto the coverslips by incubating them in the respective antibiotic-free human cell media for at least 2 h before starting the experiment. Samples were infected as mentioned above at MOI 200 for 30 min. Next, samples were washed twice with PBS, before fixation with 4% paraformaldehyde (PFA) 20 min at room temperature (RT). The PBS was washed away with two dH_2_O washes, 3 min each. The samples were stained with 1% osmium tetroxide (O_*s*_O_4_, Electron Microscopy Sciences, Hatfield, PA, United States) for 30 min, followed by two dH_2_O washes for 3 min, and dehydrated as follows: 50, 70, and 96% ethanol dehydrations, each 3 min, were performed before two 5-min dehydrations in 100% ethanol. Last, the dehydration was finalized with an overnight incubation of samples in ≥99.9% hexamethyldisilazane (Sigma). Coverslips were then plated onto specimen studs with carbon stickers (Ted Pella Inc., United States). Samples were stored in a dehumidifier chamber at RT until imaging with Zeiss Orion Nanofab HIM. Acceleration voltage of approximately 30 kV and an aperture of 10 μm were used at 25° tilt. The spot size varied between 6 and 7, with the ion current in the range of 0.09–0.3 pA. Since the samples were non-conductive, the flood gun charge compensation was utilized. Images were taken using line averaging of 32 with 0.5–2 μm dwell time. In total, 20 infected cells from two separate experiments were imaged and analyzed for *B. burgdorferi* invasion.

### *Borrelia burgdorferi* Infection Assay and Wheat Germ Agglutinin Staining

Analyzing the infectivity of *B. burgdorferi* with the human cells was performed at time points 24, 48, 72, 96 h, 7 and 9 days using MOIs 10, 20, and 40. In each experiment, 30,000 cells/well in a 12-well plate were seeded and incubated overnight before the infection with *B. burgdorferi*.

At each time point, cells were fixed, stained and immunolabeled as previously described (Thammasri et al., 2013). All steps were performed at RT and in the dark after the first staining step. Briefly, the samples were washed twice with PBS after the fixation step mentioned above. Free aldehydes were blocked with 0.15% glycine/PBS solution for 10 min, and unspecific binding was blocked with 2% BSA (Sigma)/PBS solution for 20 min. Cell membranes were stained with wheat germ agglutinin (WGA) with Texas Red-X conjugate (Invitrogen) for 20 min. WGA was used at a concentration of 10 and 5 ng/ml for SW1353 and BJ cells, respectively. After staining, cells were washed twice with PBS for 5 min. Coverslips were mounted onto microscopic slides (Thermo Scientific) with Antifade Prolong Gold with DAPI (Sigma). Leica TCS SP8X Falcon confocal microscope with the 63 × glycerol objective, PMT detector for the 405 nm, and HyD detectors for the 488 and 555 nm excitation wavelengths were used to image the samples. Optimal exposure, gain, and intensity were adjusted with Las X software (Leica) before taking the images. A human cell was considered infected if *B. burgdorferi* was attached to or inside the cell. A total of 300 human cells were counted from three separate experiments.

### Human Cell Viability Assays

The viability of *B. burgdorferi* infected human cells at various time points (24, 48, 72, and 96 h) was examined using two separate methods. Uninfected cells were used as a positive control, while 1 μM Staurosporine (S4400 staurosporine from Streptomyces sp., Sigma) was used to stimulate human cell death. MOIs of 20, 40, and 200 were used to infect 30,000 cells in 12-well culture plates (Nunc). After each time point, samples were trypsinized with 0.05% trypsin/EDTA solution (Gibco) and counted with Trypan Blue exclusion method, see below. To further verify the viability of the cells, 100 nM MitoTracker Red CMXRos (MT, Invitrogen) was used to stain viable mitochondria as instructed by the manufacturer. Samples were prepared for both flow cytometry and confocal microscopy analysis. After the MT staining, the microscopy samples were fixed and mounted onto microscopic slides as mentioned above, while the samples required for flow cytometric analysis were trypsinized and plated onto round bottomed 96-well plate (Corning). Guava^®^ easyCyte 8HT benchtop flow cytometer (Millipore) with blue and red lasers on, 5-decade acquisition, a 3,000 threshold for forward scatter and the 583/26 nm filter, was used to analyze samples. Guava InCyte 3.0 software was used to acquire either 10,000 events or 3 min of event collection, as well as in analyzing the data. Regions for cells and *B. burgdorferi* were drawn in the scatter plots, and the fluorescence gate was adjusted for cells with unstained controls. Each experiment was performed three times with triplicate samples.

### *Borrelial* Survival

To examine the possibility of *B. burgdorferi* to survive and remain viable after being internalized by a human cell, an infection procedure was performed using MOI 200 for 24 and 72 h. After the time points, external bacteria were removed using an acid wash protocol as previously described with modifications (Kameyama et al., 2007). Briefly, the cells were washed three times with cold PBS, before the samples were subjected to a cold acid wash (0.2 M glycine, 0.15 M NaCl, pH 3) for 30 s. Then three 30 s cold PBS washes were performed. The cells were detached from the plates by trypsinization for 5 min at +37°C, and collected into Eppendorf tubes using antibiotic-free media. The viability of the human cells was examined with Trypan Blue. For the Trypan Blue assay, the cells were pelleted (200 g, 5 min, Thermo Scientific MicroCL 17, Germany) and washed twice with RT PBS. After the second wash, the pellet was resuspended into 3 ml of *B. burgdorferi* media (BSK II) and a 10-μl sample containing Trypan Blue was taken into a C-chip hemocytometer for microscopical analysis to ensure that both the human cells were not damaged after the acid wash and that no free *B. burgdorferi* was left in the samples. The pellets were incubated in BSK II containing culture tubes at +37°C for a maximum of 6 weeks. At week 4, the media in the samples was changed by centrifugation (1,000 × *g* for 15 min). Each week, the samples were examined for *B. burgdorferi* growth by taking a 10 μl sample for fluorescence microscopic analysis. *B. burgdorferi* growth was determined if the samples had multiple healthy looking and motile spirochetes. Leica DM5500 fluorescence microscope with ×20 and ×40 objectives and 488 nm filter setup were used in visualizing and imaging the samples. The experiments were carried out in triplicates and repeated three times.

### Transmission Electron Microscopy

In order to visualize the intracellular location of *B. burgdorferi*, transmission electron microscopy (TEM) was utilized at 24 h and 9-day time points with MOI 200 (10 cm dish). Samples were prepared for immunolabeling on frozen thin sections as described previously (Huttunen et al., 2014). Samples were washed twice with RT PBS before fixation with 4% PFA, 0.1% glutaraldehyde in 0.1 M phosphate buffer for 10 min. After the fixation, the cells were gently scraped from the culture plates and further fixed for a maximum of 1 h. The cells were pelleted in a swing-out rotor at 2,700 × *g* for 10 min RT (Heraeus Megafuge 1.0 R, Germany). Rabbit anti-GFP primary antibody (Invitrogen) was used in immunolabeling *B. burgdorferi* with 1:100 dilution. Protein A gold (10 nm, 1:200) was used to visualize *B. burgdorferi* as described previously (Huttunen et al., 2014). JEOL JEM1400 transmission electron microscope was utilized in imaging the samples.

### Co-localization and Green Fluorescent Protein Signal Analyses With Immunolabeling Procedure

For a co-localization study between *B. burgdorferi* and lysosomes, 30,000 cells on coverslips in 24-well plates were infected with MOI 40 of *B. burgdorferi* for 24 h and 9 days. The cells were fixed, and the membranes were stained with WGA as mentioned above. After the WGA staining, two 5-min PBS washes were performed, and the cell membrane was permeabilized with Triton-X100 solution (0.1% Triton X-100 (Sigma), 0.01% NaN_3_ (Sigma), 2% BSA in PBS) for 20 min. The H4B4 mouse anti-human Lamp-2 primary antibody (Developmental Studies Hybridoma Bank, United States) was diluted to 1:50 in the Triton solution, and the samples were incubated for 1 h. Afterward, three PBS washes, 5 min each, was performed. Alexa fluor goat anti-mouse IgG 633 secondary antibody (Invitrogen) was used at 1:200 dilution in the Triton solution and incubated for 30 min. Excess secondary antibody was washed with PBS, three times for 5 min. The samples were mounted with Antifade Prolong Gold with DAPI. Nikon A1R confocal microscope with Galvano scanning, 60× objective and 405 nm (DAPI), 488 nm (GFP), 561 nm (WGA), and 638 nm (Lamp2) lasers were used in the co-localization experiment. Stacks with 0.2 μm steps of 30 cells in total were taken for the co-localization analysis.

For the GFP signal analysis, ×40 objective with the same excitation wavelengths as in the co-localization analysis, except the 638 nm laser, were used. From randomly selected portions of the samples, 30 images of cells were taken from the middle section of stacks (middle of the cell). Both experiments were repeated three times.

### Pleomorphic Form Analysis

During the *B. burgdorferi* infection assay, it was noted that there were clear differences in *B. burgdorferi* forms between the infections of the two human cell lines. Therefore, confocal imaging (Nikon A1R microscope with Galvano scanning, ×60 objective with 405, 488, and 561 nm lasers) was utilized to analyze the different pleomorphic forms of *B. burgdorferi* found in the infected samples. In addition, *B. burgdorferi* was subjected to BJ (MEM) and SW1353 (L-15) medias alone for 96 h and analyzed daily with a fluorescence microscope (Leica DM5500). In total, 300 *B. burgdorferi* associated with the human cells were counted from three separate experiments. The included forms (Meriläinen et al., 2015) and their inclusion/exclusion criteria are stated in Table 1 below.

**TABLE 1:** Pleomorphic forms of *Borrelia burgdorferi* and their characteristics.

**Form**	**Description**	**DNA**	**GFP**	**Size**
Spirochete	Corkscrew or any other clear spirochete form but in a variety of shapes [e.g., looped, ring (Miklossy et al., 2008), V-shaped etc.]	Yes	Yes	10–30 μm
Bleb	Spirochete with clear blebbing, can have several blebs, OR a detached bleb.	Yes/No	Yes	0.8–1.7 μm
Round body (RB)/coiling RB	Full RB or a forming RB that can include a “tail”	Yes	Yes	Full RB 2.4–3.2 μm Coiling RB >1.7 μm
Aggregate	Cluster of 10 or more bacteria, can include any one or all of the abovementioned pleomorphic forms	Yes	Yes	Varies, must have 10 or more bacterial cells
Damaged	Form/shape difficult to define into any of the abovementioned forms. Must lack either a DNA or GFP signal. Spheres are smaller than 0.8 μm			

*GFP, green fluorescent protein.*

### Image and Statistical Analyses

All confocal images were analyzed using ImageJ software (Schindelin et al., 2012). Brightness and contrast were adjusted, Gaussian blur filter with Sigma radius 0.5–0.7 was added, and stacks were combined with z-projection using max intensity to create a single image.

The co-localization analysis was performed in ImageJ using the JaCoP plugin (Bolte and Cordelières, 2006). Before starting the analysis, Intermodes threshold was used for the stacks with additional manual adjustments. The analysis was executed using Mander’s coefficient and Costes’ randomization with 100 rounds. Samples with more than 5% co-localization were considered as co-localized if their Costes’ *p*-value was ≥0.95 (Costes et al., 2004). The representative images were made using the Intermodes threshold.

The HIM images were adjusted for brightness and contrast using ImageJ.

Two-tailed, unequal variance Student’s *t*-test was performed in Microsoft Excel. Statistical significance was considered for samples with a *p-value* of ^∗^ ≤ 0.05 or ^∗∗^ ≤ 0.005.

## Results

### *Borrelia burgdorferi* Formed Coiled Structures During Entry Into Human Cells

**FIGURE 1:**
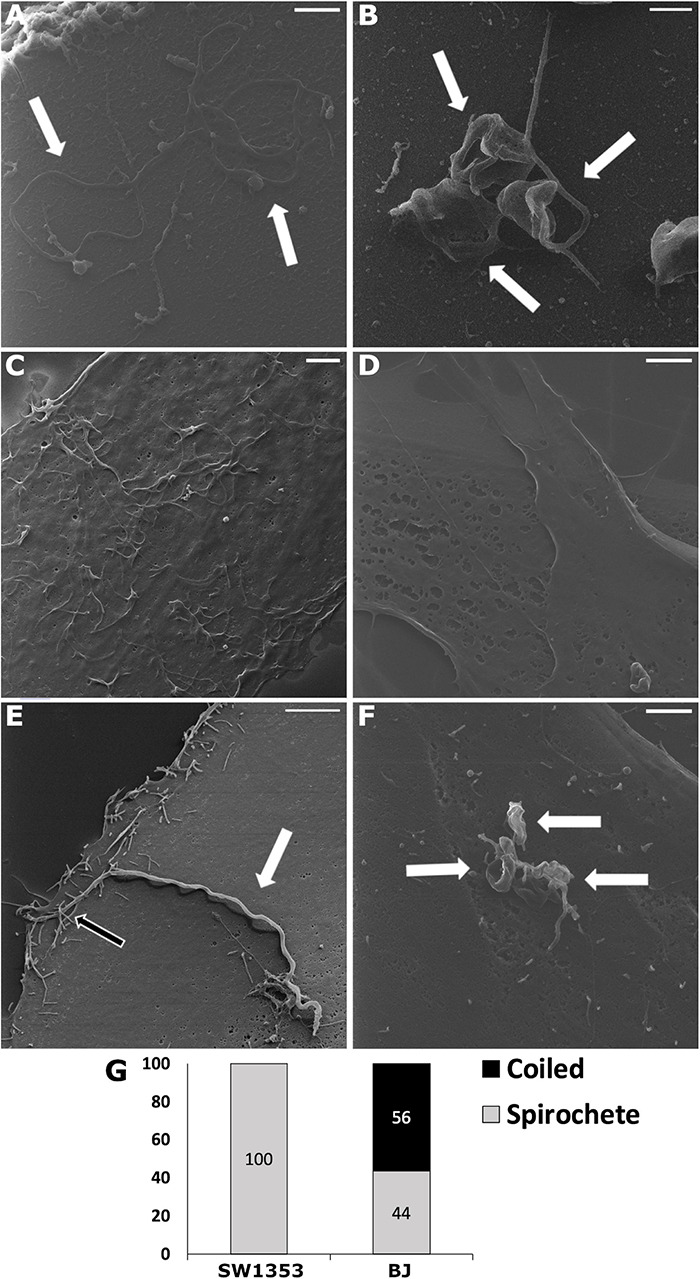
Upon infection, human cell lines demonstrated *Borrelia burgdorferi* forms differently. Helium ion microscopy (Zeiss Orion Nanofab) images of *Borrelia* spirochetes **(A)** and round bodies **(B)**, as well as, uninfected chondrosarcoma (SW1353) **(C)** and dermal fibroblast (BJ) **(D)** cells. **(E,F)** *Borrelia* spirochetes and coiled forms invaded SW1353 and BJ cells, respectively, at 30 min post-infection. **(G)** A total of 20 infected human cells were counted and the different *B. burgdorferi* forms were analyzed. Graph presents spirochete and coiled forms attached to SW1353 and BJ cells in percentages. White arrows indicating *Borrelia*, while the black arrow points to cellular interactions with the bacterium. Scale bars **(A–C)**: 1 μm, **(D–F)**: 2 μm. Representative images from two separate experiments.

With helium ion microscopy the surfaces of biological samples can be examined without any additional metallic coating procedures of samples, which might affect and distort results. Hence, HIM was applied to detect the cell attached *Borrelia* at an early time point (30 min post-infection) onto both human dermal fibroblast and chondrosarcoma cells. To the best of our knowledge, this is the first time HIM has been utilized in analyzing early cellular invasion by *B. burgdorferi*. In the control images, multiple spirochetes, and a cluster of three RBs, respectively, demonstrate the shapes and sizes of *B. burgdorferi* (Figures 1A,B). Carcinoma cells have been shown to exhibit lamellipodia and invadopods as adhesion and invasion structures (Lorenz et al., 2004). Here, the uninfected carcinoma cell line SW1353 displayed cell surface extensions in higher amounts than the BJ cell line (Figures 1C,D). It was considered that the effect was due to the invasive nature of the cell line, since BJ cells were not a cancer cell line. A total of 20 human cells were counted and analyzed for *B. burgdorferi* entry from the infected samples. Only spirochetes were located on top of the infected SW1353 cell (Figures 1E,G), while both spirochetal and coiled forms were observed on the BJ cells (Figures 1F,G) 30 min post-infection. The white arrows in Figure 1 point to *B. burgdorferi* in the infected samples, as well as in the controls. *B. burgdorferi* was found interacting with cell surface constructs of the SW1353 cells as indicated by the black arrow (Figure 1E).

### *Borrelia burgdorferi* Infected Human Cells Differently

**FIGURE 2:**
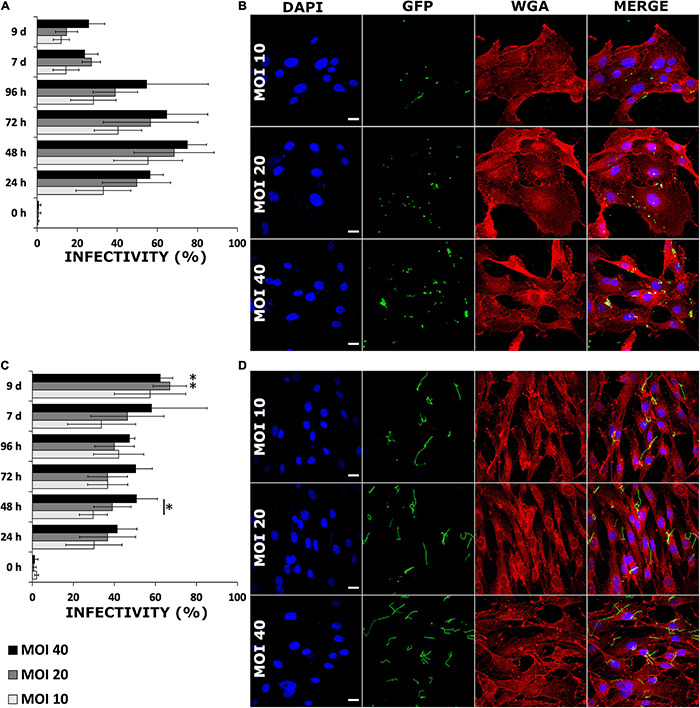
*Borrelia burgdorferi* infected human cells in a dose dependent manner without losing infectivity even after 9 days. SW1353 **(A,B)** and BJ **(C,D)** cells were infected with *B. burgdorferi* strain GCB726 with green fluorescent protein (GFP) (*B. burgdorferi*). A cell was considered infected when *B. burgdorferi* was attached to or inside a cell. Three different multiplicity of infections (MOIs) (10, 20, and 40) were examined over 9 days. In total, 300 cells were counted from three replicate experiments (*n* = 300). Standard deviation of means from three repeated experiments. Statistical significance with paired independent Student’s *t*-test (*p ≤ 0.05) was performed for both MOIs in a time point (line with asterisk), as well as, between 24 h and the rest of the time points (asterisk only). Representative confocal micrographs from the 48 h time point for infected SW1353 **(B)**, and BJ **(D)** cells. The nucleus (DAPI) in blue, *B. burgdorferi* (GFP) in green, the cell membrane wheat germ agglutinin (WGA) in red and a merged image of all three channels. Scale bars 20 μm.

Two human cell lines, chondrosarcoma (SW1353) and a dermal fibroblast (BJ), were infected with GFP-mutated strain of *B. burgdorferi* in order to investigate the infectivity of the bacterium in these cells. During a 9-day time period, the differences between the infections of the different cell lines was evident. An overall trend in the infected SW1353 cells was that the amount of cell associated bacteria increased until 48 h (Figure 2A). Although infectivity increased in a dose-dependent manner inside a time point, the increase was not significant (*p* ≤ 0.05) (Figures 2A,B). Conversely, the infectivity of *B. burgdorferi* with the BJ cell line increased gradually reaching a peak at day 9 (Figure 2C). Similarly to SW1353 cells, infectivity increased in a dose dependent manner with a significant difference between MOIs 10 and 40 (*p* ≤ 0.05) at 48 h (Figures 2C,D). Furthermore, at 9 days post-infection, there was a significant increase in the infectivity of BJ cells with MOIs 20 and 40 when compared with 24 h. Noticeably, even at 9 days post-infection, *B. burgdorferi* was not cleared from either cell line.

Since the infectivity of *B. burgdorferi* with these two human cell lines demonstrated to be different, an additional experiment measuring the GFP signal from the infected samples at 24 h and 9 days was performed. Using a 40× objective, a total of 30 images of clusters of cells with a confocal microscope were taken, each from the middle of the cells, and analyzed for GFP signal intensities. The mean values for each image were combined and averaged, and a comparison between the time points was carried out (Supplementary Figure 1). There was a significant decrease (*p* ≤ 0.005) of GFP signal in the later time point in the infected SW1353 cells, while, the GFP signal intensities did not significantly vary in the BJ cell line between the time points (Supplementary Figure 1A). The top two rows in Supplementary Figure 1B illustrate the SW1353 cell line for 24 h and 9 days, respectively. The bottom two rows in Supplementary Figure 1B represent BJ cell line. As demonstrated in the GFP images, more *B. burgdorferi* were visible in the BJ samples, than in the SW1353 cells.

### Human Cells Remained Viable After *Borrelia burgdorferi* Infection

**FIGURE 3:**
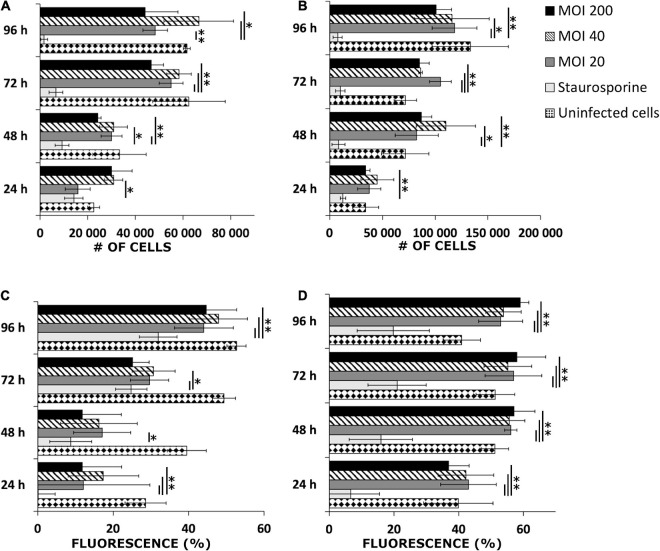
Human cells remained viable after *B. burgdorferi* infection. Representative graphs of viable SW1353 **(A)** and BJ **(B)** cells infected with *B. burgdorferi* at four different time points and cells counted using Trypan Blue stain. Experiments were performed in triplicates and repeated three times. Uninfected cells were negative control, while staurosporine induced apoptotic cells were positive controls for death. MOIs 20, 40, and 200 were used. MitoTracker^TM^ Red CMXRos stain for live mitochondria was used to further verify the viability of the infected cells from three separate experiments. Flow cytometry was used to analyze the samples. A graphical illustration of the fluorescence signal in percentages (%) gathered from SW1353 **(C)** and BJ **(D)** cells, respectively. The same controls and MOIs were used as in the Trypan Blue experiments above. Error bars of standard deviations. Statistical significance was performed with Student’s *t*-test (*p ≤ 0.05; **p ≤ 0.005).

An investigation into cellular viability after *B. burgdorferi* infection was performed. Both human cell lines were infected with three different MOIs (20, 40, and 200) and then counted after four different time points using Trypan Blue. Both infected cell lines grew in a similar manner to the uninfected cells throughout the 96 h (Figures 3A,B). In the infected SW1353 cells, MOI 40 samples had significant replication (^∗^*p* ≤ 0.05; ^∗∗^*p* ≤ 0.005) in each time point when compared with the staurosporine induced apoptotic cell control (Figure 3A). Furthermore, after the first 24 h, each MOI varied significantly from the positive control. Similarly, the infected BJ cells multiplied significantly during the four time points. However, in the BJ cells, MOI 200 samples had significant growth (*p* ≤ 0.005) in each time point, with significant growth for MOIs 20 and 40 starting at 48 h (Figure 3B). Hence, both cell lines remained viable even with high doses of *B. burgdorferi*.

In addition, viable mitochondria were stained and analyzed with a flow cytometer to further confirm the viability of the human cells. MOI 20 differed significantly in each of the four time points with the infected SW1353 cells when compared with the positive control (Figure 3C). Quite markedly, a very strong significance (*p* ≤ 0.005) in the SW1353 samples was found at 24 and 96 h timepoints for each MOI. In a similar manner, the infected BJ cells demonstrated significantly strong differences between the positive control and the MOIs at each time point (Figure 3D). Both cell lines had viable mitochondria even with high MOIs throughout the experimental time points. Hence, it was deduced that the human cells remained viable 96 h post-infection.

Representative dot plots of the flow cytometry analysis, and confocal microscopy images of mitochondria-stained samples from the 96-h time point can be found in Supplementary Figure 2. The unstained controls were used to adjust the fluorescence signal. The cell controls were used to denote the viable cell populations in the middle of the plots (red circles), while *B. burgdorferi* is located in the red squares on the left. The infected samples exhibited similar cell patterns to the negative controls in both cell lines (Supplementary Figures 2A,B). The confocal micrographs represent the viability of the infected SW1353 and BJ cells, respectively, as seen by staining of the viable mitochondria (MT) in Supplementary Figures 2C,D. Only the highest MOI (200) is presented.

### *Borrelia burgdorferi* Could Be Regrown After Internalization Into Human Cells

**FIGURE 4:**
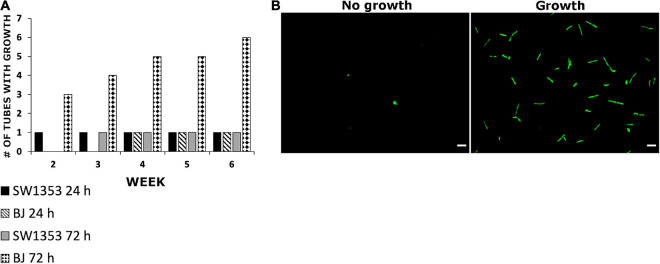
*Borrelia burgdorferi* was able to regrow after being internalized into human cells. **(A)** SW1353 and BJ cells were infected with *B. burgdorferi* for 24 and 72 h, respectively. After each time point, the cells were thoroughly washed with PBS and treated with an acid wash (0.2 M glycine, 0.15 M NaCl, pH 3) for 30 s in order to get rid of any external bacteria. The cells were then placed into BSK II media for 6 weeks and observed weekly for borrelial growth using fluorescence microscopy. Growth was considered when samples contained multiple healthy looking and motile spirochetes **(B)**. The experiments were performed in triplicates and repeated three times, thus, totaling nine tubes for each cell line and time point. Scale bars 30 μm.

During the cell survival experiments, the question of whether *B. burgdorferi* remained viable after internalization into the human cells arose. In order to study borrelial survival, the cell lines were infected with *B. burgdorferi* and, after 24 and 72 h post-infection, the washed cell pellets were placed in BSK II media for 6 weeks. By using a fluorescence microscope, *B. burgdorferi* growth was considered positive once a sample contained multiple motile spirochetes, while samples lacking growth had negligible fluorescence signal. During the 6-week growth period, *B. burgdorferi* was observed to grow in total only in one out of nine samples from the SW1353 cells from triplicate experiments at both time points (Figure 4). Also, from the BJ cell samples, *B. burgdorferi* was found growing from one out of nine test samples from the 24-h time point. However, a total of six out of nine from the 72-h time point samples had grown by the end of the 6 weeks (Figure 4). The data indicated that *B. burgdorferi* was capable of growth after infection for several days, though growth was not observed in all samples.

### *Borrelia burgdorferi* Was Intracellular Even After 9 Days Post-infection

**FIGURE 5:**
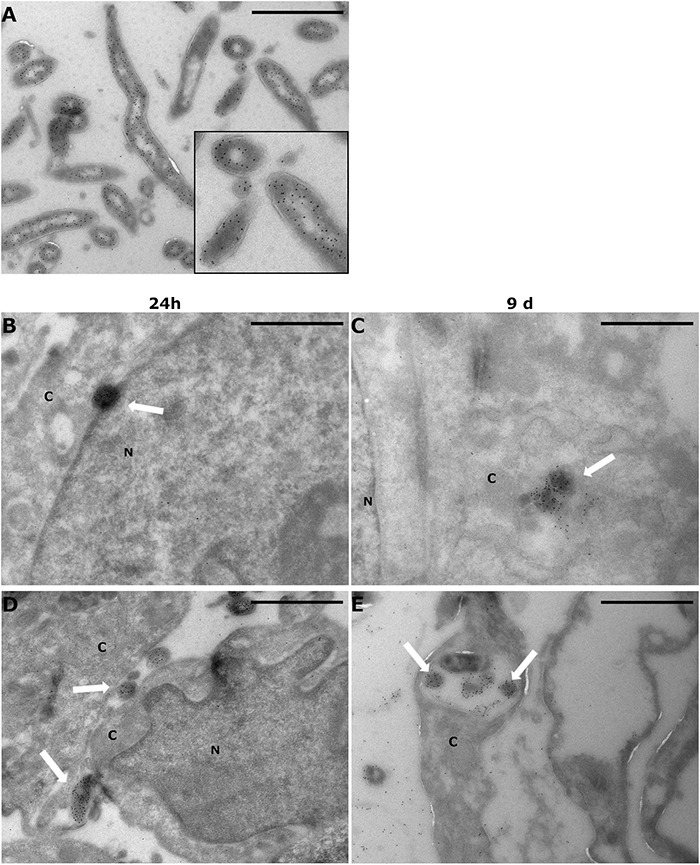
*Borrelia burgdorferi* was found inside human cells even after 9 days post-infection. SW1353 and BJ cells were infected with *Borrelia* GCB726 strain with GFP for 24 h and 9 days. Infected cells were prepared for cryo-electron microscopy with GFP immunolabeling using anti-GFP antibody and Protein A-gold particles (diameter 10 nm). Transmission electron microscopy (JEOL JEM1400) was utilized in visualizing the samples. **(A)** *Borrelia* control with a zoomed image (black box), **(B,C)** SW1353 samples at 24 h and 9 days, respectively, and at the bottom the infected BJ cells **(D,E)**. White arrows indicate *Borrelia* in the infected samples. Cytoplasm (C) and nucleus (N) of the cells are indicated. Scale bars 1 μm.

To investigate the intracellular location of *B. burgdorferi*, cryo-EM was employed. Twenty-four hours and 9 days were considered useful time points for visualizing the expected changes in *B. burgdorferi* infectivity, which were evident in the *B. burgdorferi* infection assay mentioned above. The analysis of the GFP and Protein A-gold immunolabeled samples indicated that *B. burgdorferi* was present in the cells even at 9 days post-infection (Figures 5C,D). The immunolabeled *B. burgdorferi* (Figure 5A) could be found both inside and outside the human cells at both time points. The intracellular locations varied from close to the nucleus, in the cytosol, attached to the plasma membranes, and inside a cellular vesicle (Figures 5B–E).

### *Borrelia burgdorferi* Was Not Targeted to Lysosomes

**FIGURE 6:**
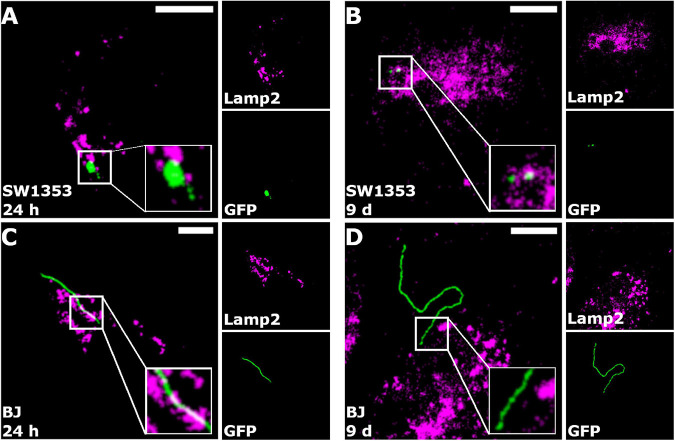
*Borrelia burgdorferi* did not co-localize with lysosomes. Merged representative images of SW1353 **(A,B)** and BJ **(C,D)** cells infected with *B. burgdorferi* at 24 h and 9-day time points, respectively. *Borrelia* fluorescence green, lysosomes in magenta, and co-localized pixels appear white. Zoomed images are shown in the white boxes at the bottom right corners of the merged images. As indicated in the merged images, co-localization of *B. burgdorferi* with lysosomes was not observed. Intermodes threshold was applied to the images before analysis with JaCoP plugin in ImageJ. Scale bars 10 μm.

In an effort to determine a possible processing pathway of *B. burgdorferi* inside the human cells, co-localization analysis of GFP and lysosomes was carried out. The analysis was performed at 24 h and 9 days, with a total of 30 cells from three separate experiments. Only cells with a co-localization value of over 5% were considered as co-localized if the Costes’ *p*-value was higher than 0.95 (Costes et al., 2004). In Figure 6, the representative images of the co-localization analysis are presented. Lysosomes are represented in magenta, GFP (*Borrelia*) in green, and the co-localized pixels are indicated in white. As displayed in the zoomed images (white boxes) of each figure, very little co-localization occurs with *B. burgdorferi* and the lysosomes (Figures 6A–D). This indicated that *B. burgdorferi* was not targeted to lysosomes and could avoid degradation by lysosomal enzymes. However, specifically in the SW1353 cell samples (Figures 6A,B), it can be seen that *B. burgdorferi* is not in its spirochetal forms but rather in distinguishable RB or coiled shapes.

Table 2 demonstrates the mean values for both co-localized and non-co-localized pixels for the GFP and Lamp2 signals with the number of cells (n) next to each cell line. Table 2 indicates that there are only two SW1353 cells with an average of 8.5% co-localization of lysosomes with *B. burgdorferi*, and only four BJ cells with 16.4% co-localization value at 24 h. At day 9, the co-localized SW1353 cell numbers increased to 13 with an average co-localization value of 17.5%. Contrary to SW1353 cells, the number of BJ cells with co-localized lysosomes with *B. burgdorferi* dropped to zero at day 9.

**TABLE 2:** *Borrelia burgdorferi* did not co-localize with lysosomes—the number of human cells (n) with average percentages of co-localization between *Borrelia* and lysosomes, and Costes’ *p*-value, at 24 h and 9 days post-infection.

	**Co-localization (>5%)**		***p*-value**	**No co-localization (<5%)**		***p*-value**
24 h	SW (*n* = 2)	8.5	100	SW (*n* = 28)	0.32	53.4
	BJ (*n* = 4)	16.4	100	BJ (*n* = 26)	0.55	50.2
9 days	SW (*n* = 13)	17.5	100	SW (*n* = 17)	0.44	43.6
	BJ (*n* = 0)	0	0	BJ (*n* = 30)	0.74	61.2

### Human Cell Lines Displayed *Borrelia burgdorferi* Pleomorphic Forms Differently

**FIGURE 7:**
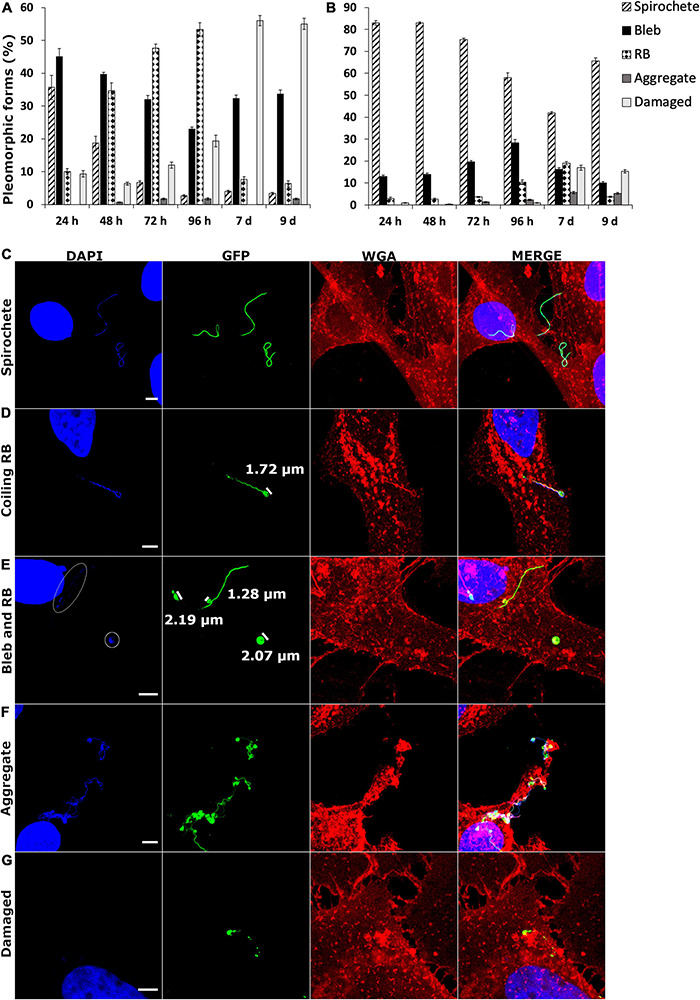
*Borrelia burgdorferi* infection resulted in different formation of pleomorphic forms between the two human cell lines. SW1353 **(A)** and BJ **(B)** cells were infected with *B. burgdorferi* at several time points. A total of 300 *B. burgdorferi* from three experiments were calculated and characterized into spirochete, bleb, round body (RB), aggregate, and damaged categories (the different category definitions can be found in Table 1 in the “Materials and Methods” section). Different pleomorphic forms in the two human cell lines were observed. **(C)** Three spirochetes are evident, while in panel **(D)**, a coiling RB is visible with the coiling head measuring 1.72 μm. Comparison with a bleb (1.28 μm) to an RB (approximately 2.0 μm) are demonstrated in panel **(E)**. DNA signal from the RB on the right, as well as, from the bleb are indicated in white circles. An aggregate is represented in panel **(F)**, while panel **(G)** indicates a damaged *B. burgdorferi* with a clear lack of bacterial DNA. DNA is visualized in blue (DAPI), *B. burgdorferi* in green (GFP) and the cell membrane in red (WGA). Merged images of the channels are provided. Scale bars 5 μm.

During the *B. burgdorferi* infection analysis, the two human cell lines demonstrated a variety of pleomorphic forms. Therefore, an analysis of 300 *B. burgdorferi* from each cell line and time point was performed from confocal images of GFP-labeled *B. burgdorferi*. The different pleomorphic forms of *B. burgdorferi* were divided into spirochetes, blebs, round bodies, aggregates, and damaged (Meriläinen et al., 2015). The specific requirements for each category can be found in Table 1 in the “Materials and Methods” section. In Figures 7A,B, the division of each category in percentages for infected SW1353 and BJ cells, respectively, are shown. The longer the time point, the more pleomorphic forms were identified from both cell lines. Nonetheless, while both cell lines exhibited each of the pleomorphic form, there was a clear difference between the cell lines. SW1353 cells displayed more blebs and RBs during the first 96 h, with an abundance of damaged forms at 7 and 9 days post-infection (Figure 7A). For the SW1353 cell samples, there was a steady increase of RBs and a decrease of blebs during the first 96 h. A sudden drop of RBs and an increase of damaged forms at 7–9 days occurred in the SW1353 cells. Hence, the parental spirochetal form changed to the other pleomorphic forms from the beginning, with less than 40% of the population being spirochetes at 24 h (Figure 7A). In the infected BJ cells, on the other hand, the spirochetal forms remained the dominant shape throughout the time points with over 50% still as spirochetes at 9 days post-infection (Figure 7B). Although there was a somewhat steady increase of blebs, there were only less than 30% of blebs in the whole population at 96 h (Figure 7B). At 7 days post-infection, there were a variety of both pleomorphic and damaged forms visible; however, both blebs and RBs, as well as, the damaged forms, accounted for only less than 20% of the forms observed in the infected BJ cells (Figure 7B). Interestingly, the number of both blebs and RBs decreased, while the number of spirochetes increased at 9 days post-infection (Figure 7B). Furthermore, aggregates steadily increased in the infected BJ cells throughout the analyzed time points. Therefore, the utilization of pleomorphic forms during infection of different human cell lines was regarded as a mechanism to aid in survival and/or persistence for *Borrelia*.

Previous analysis of *Borrelia* survival in MEM media has indicated the maintenance of spirochetal forms (Murgia and Cinco, 2004), while a separate investigation of RPMI-1640 media indicated the induction of RBs, blebs, and damaged spirochetes (Meriläinen et al., 2016). Therefore, an analysis of *B. burgdorferi* in BJ (MEM) and SW1353 (L-15) media alone for 96 h was performed (data not shown). Mostly spirochetes were seen in MEM, but in L-15, a similar pattern to the formation of especially damaged *Borrelia* as in Figure 7A was observed. However, as the included *Borrelia* from the infected cell culture samples had to be either intracellular or attached to the human cells, the formation of different pleomorphic forms could not be solely explained by the unfavorable conditions of the L-15 media even at 9 days. Hence, the results in Figure 7 are considered valid and due to the differences in the human cells and not in the cell media.

In Figures 7C–G representative images of spirochetes, coiling RB, blebs, and RBs, aggregates and damaged forms, respectively, can be observed. As can be seen, the RBs in Figures 7D,E have a DNA signal, as well as, a GFP signal, and are wide enough to be categorized as RBs. While the bleb in Figure 7E has DNA but lacks the width in the bulge to be considered as an RB. The weak DNA signals in Figure 7E have been circled for better visualization. An aggregate with a collection of 10 or more bacterial cells is visualized in Figure 7F. A damaged *B. burgdorferi* with a clear lack of DNA signal is exemplified in Figure 7G.

## Discussion

*Borrelia burgdorferi* infection can lead to a multisystemic disorder affecting predominantly the skin, joints, and the nervous system. Yet, it remains uncertain how the initial infection can sometimes lead to prolonged distress in patients. *Borrelia* has been shown to infect non-immune cells, such as human foreskin fibroblasts (Georgilis et al., 1992; Klempner et al., 1993), human primary synovial cells (Girschick et al., 1996), human umbilical vein endothelial cells (HUVECs) (Comstock and Thomas, 1989, 1991; Szczepanski et al., 1990; Ma et al., 1991; Livengood and Gilmore, 2006), human neural cells (Strnad et al., 2015), as well as human neuroglial (Rittig et al., 1992; Livengood and Gilmore, 2006; Williams et al., 2018), and neuroblastoma cells (Thomas et al., 1994; Strnad et al., 2015), among others. The bacterium’s ability to invade non-phagocytic cells has been suggested as one mechanism for immune evasion (Ma et al., 1991; Klempner et al., 1993; Girschick et al., 1996; Embers et al., 2004; Livengood and Gilmore, 2006; Wu et al., 2011; Naj and Linder, 2015). Table 3 summarizes studies of different mammalian cell lines infected with *Borrelia* and the adverse outcomes of the infection. In this study, we investigated the infection of two non-phagocytic human cell lines, normal dermal fibroblast (BJ), and chondrosarcoma (SW1353) cells, by *B. burgdorferi*, and the outcome of the infection for both the human, as well as, the bacterial cells.

**TABLE 3:** Synopsis of previous studies of borrelial infections in a variety of mammalian cell lines.

**Host cell (name)**	***Borrelia* inoculum**	**Host cell viability**	***Borrelia* viability**	**Borrelial invasion mechanism**	**Borrelial location on/inside the cell**	**Other**
HUMAN Endothelial (HUVEC) (Comstock and Thomas, 1989, 1991; Thomas and Comstock, 1989; Szczepanski et al., 1990; Ma et al., 1991; Thomas et al., 1994; Livengood and Gilmore, 2006) Epithelial (HeLa) (Isaacs, 1994)	50 × 10^6^ (Thomas and Comstock, 1989; Comstock and Thomas, 1991; Thomas et al., 1994) MOI 40 (Livengood and Gilmore, 2006) MOI 300 (Isaacs, 1994)	After 4 h (Comstock and Thomas, 1989) and 7 days (Livengood and Gilmore, 2006) of co-culture host cells remained viable (analyzed with trypan blue)		Attachment to the host cell along the length or from the tips of the spirochete (Thomas and Comstock, 1989; Thomas et al., 1994)	Apical surfaces/intercellular spaces/beneath cell monolayer (Szczepanski et al., 1990) and through cytoplasm (Comstock and Thomas, 1991); host membrane bound intracellular bacteria as seen with EM (Comstock and Thomas, 1989)	Attachment was time, dose (Thomas and Comstock, 1989; Szczepanski et al., 1990) and temperature dependent (Isaacs, 1994); internalization peaked at 48 h and was inhibited with 1 μg/ml of cytochalasin D (Ma et al., 1991); attachment could be substantially (60%) inhibited with glycosaminoglycans (Isaacs, 1994)
Primary fibroblast (Georgilis et al., 1992; Klempner et al., 1993; Rittig et al., 1996; Wu et al., 2011)	MOI 10 (Rittig et al., 1996; Wu et al., 2011) 4/40/400 × 10^6^ (Klempner et al., 1993)	Host remained viable with internalized bacteria (Klempner et al., 1993; Wu et al., 2011)	Internalized *Borrelia* survived 5 days (Klempner et al., 1993) and 14 days (Georgilis et al., 1992) treatment of ceftriaxone, and 5 h treatment with gentamicin (Wu et al., 2011)	Invaginations and extensions into host cytoplasm without any apparent perturbations from the fibroblasts (Klempner et al., 1993; Rittig et al., 1996)	Apical surfaces (Klempner et al., 1993)	Attachment was dose dependent (Klempner et al., 1993); long term internalization and maintenance of viable *Borrelia* by host cells (after 28 days of co-culture) (Wu et al., 2011)
Primary synovial (Girschick et al., 1996)	MOI 100 (Girschick et al., 1996)	Host remained viable in co-culture after 8 weeks (Girschick et al., 1996)	Internalized *Borrelia* survived 9 days treatment of ceftriaxone for 8 weeks (Girschick et al., 1996)	Engulfment without enwrapping of host membrane or phagosome interaction as visualized with EM (Girschick et al., 1996)	After 5 days of co-culture, spirochetes in the host cytoplasm without signs of degradation (Girschick et al., 1996)	Long term internalization and maintenance of viable *Borrelia* by host cells (after over 8 weeks of co-culture) (Girschick et al., 1996)
Neuroglia (H4 and HS-683) (Thomas et al., 1994; Livengood and Gilmore, 2006; Williams et al., 2018) Neuroblastoma (SK-N-MC) (Thomas et al., 1994), (UKF-NB-4) (Strnad et al., 2015)	MOI 10 (Strnad et al., 2015; Williams et al., 2018) MOI 40 (Livengood and Gilmore, 2006) 50 × 10^6^ (Thomas et al., 1994) MOI 100 (Williams et al., 2018)	After 5 h (Strnad et al., 2015) and 7 days (Livengood and Gilmore, 2006) of co-culture host cells remained viable (analyzed with trypan blue)	Viable *Borrelia* after 6 h in DMEM containing 1% antibiotic-antimycotic solution, but motility was lost at 8 h (Strnad et al., 2015); viable internalized *Borrelia* after 4 h incubation with gentamicin (150 g/ml) (Livengood and Gilmore, 2006)	Adherence along the spirochete (Thomas et al., 1994); coiling and conventional phagocytosis as visualized with EM (Livengood and Gilmore, 2006); coiling phagocytosis via Daam1 regulated pseudopods (Williams et al., 2018)		Attachment but no entry in 3 h of co-culture, which might be due to the antibiotics in the culture medium (Strnad et al., 2015)
Primary monocytes and macrophages (Rittig et al., 1992, 1994, 1996, 1998; Girschick et al., 1996; Hoffmann et al., 2014; Naj and Linder, 2015; Klose et al., 2021) Differentiated macrophages (THP-1) (Meriläinen et al., 2016)	MOI 10 (Rittig et al., 1992, 1994, 1996, 1998) MOI 30 (Klose et al., 2021) MOI 40 (Meriläinen et al., 2016) MOI 100 (Hoffmann et al., 2014; Naj and Linder, 2015; Girschick et al., 1996)		Internalized *Borrelia* survived 1 h treatment with 100 mg/ml kanamycin and gentamicin (Naj and Linder, 2015)	Coiled uptake and invagination into the host as visualized with EM (Rittig et al., 1994, 1996); coiling phagocytosis in 40–60% of samples vs. conventional (Rittig et al., 1996, 1998) Daam1 regulated formation of filopodia that capture spirochetes, and formation of coiling pseudopods that enwrap them (Hoffmann et al., 2014; Naj and Linder, 2015); F-actin rich pseudopods in coiling phagocytosis (Girschick et al., 1996; Meriläinen et al., 2016); conventional and coiled phagocytosis (Rittig et al., 1992, 1996; Girschick et al., 1996)	Lysosomal (Rittig et al., 1994; Meriläinen et al., 2016) degradation (Girschick et al., 1996) regulated by Rab5a and Rab22a (Naj and Linder, 2015) with some elongated spirochetes in the cytoplasm (Naj and Linder, 2015); lysosomes localized more with spirochetes than RBs (Meriläinen et al., 2016); *Borrelia* associated membranes contained tunnels, possibly formed when spirochetes had moved, which connected to ER (Klose et al., 2021)	Bacterial degradation lacked lysosomal activity (Rittig et al., 1992, 1996); pseudopods were covered with surplus pseudopods (Rittig et al., 1998); membrane ruffles and coiling but not conventional phagocytosis could be enhanced with specific chemicals (Rittig et al., 1998); cytochalasin D inhibits borrelial entry but not completely (Hoffmann et al., 2014; Meriläinen et al., 2016); *Borrelia* RBs were not internalized using coiling phagocytosis (Meriläinen et al., 2016)
Primary dendritic cell (Filgueira et al., 1996; Rittig et al., 1996; Suhonen et al., 2003)	MOI 10 (Rittig et al., 1996) 100 × 10^6^/ml (Klose et al., 2021)			Coiling (Filgueira et al., 1996) and conventional phagocytosis (Rittig et al., 1996); pseudopods coiled along the spirochete or attached to the middle of the bacterium and covered it with a broad pseudopod (Suhonen et al., 2003)	Free in the host cytosol and inside phagolysosomes (Filgueira et al., 1996) or membrane bound but not inside lysosomes (Rittig et al., 1996)	*Borrelia* infection induced IL-8 and DC maturation (Suhonen et al., 2003); bacterial degradation lacked lysosomal activity (Rittig et al., 1996)
PRIMATE Epithelial (Vero) (Georgilis et al., 1992; Hechemy et al., 1992)	200 × 10^6^ (Hechemy et al., 1992)		Internalized *Borrelia* survived 14 days treatment with ceftriaxone (Georgilis et al., 1992)	Entry at coated pit associated sites as seen with EM (Hechemy et al., 1992)	Spirochetes were free in the cytoplasm or tightly bound to host membrane (visualized with EM) (Hechemy et al., 1992)	
RODENT Mouse neuroblastoma (N2a) (Strnad et al., 2015) Rat neuroblastoma (B50)* (Rupprecht et al., 2006)	MOI 10 (Strnad et al., 2015) MOI 200 (Rupprecht et al., 2006)	After 5 h (Strnad et al., 2015) of co-culture host cells remained viable (analyzed with trypan blue)	Viable *Borrelia* after 6 h in DMEM containing 1% antibiotic-antimycotic solution, but motility was lost at 8 h (Strnad et al., 2015);	Adherence to host from the tip of the bacterium (Montgomery and Malawista, 1996; Rupprecht et al., 2006)		OspA is necessary for host cell binding; proteoglycans (heparin, heparan sulfate) aid in adherence; formation of blebs was frequent (Rupprecht et al., 2006)
Mouse macrophage (primary) (Rittig et al., 1992, 1996), (J774) (Montgomery and Malawista, 1996)	MOI 10 (Rittig et al., 1992, 1996) 100 × 10^6^/ml (Montgomery and Malawista, 1996)			Adherence to host from the tip of the bacterium (Montgomery and Malawista, 1996); conventional and coiling phagocytosis (Rittig et al., 1992), with preference toward coiling phagocytosis (>50%) (Rittig et al., 1996)	Fusion-disintegration of the lamellipodia membranes resulted in the bacteria residing in the cytoplasm (Rittig et al., 1996)	Bacterial degradation lacked lysosomal activity (Rittig et al., 1992, 1996)
Mouse fibroblast (L929) (Wu et al., 2011)	MOI 1/5/10 (Wu et al., 2011)	Host remained viable with internalized bacteria (analyzed with trypan blue) (Wu et al., 2011)	Internalized *Borrelia* survived 5 h treatment with gentamicin for 28 days (Wu et al., 2011)		Extracellular bacteria remained as spirochetes, whilst internalized occasionally were in cyst-like (RB) forms (Wu et al., 2011)	Using RT PCR, *Borrelia* gene transcripts were recovered from lysed co-cultured pellets demonstrating borrelial metabolic activity while internalized; long term internalization and maintenance of viable *Borrelia* by host cells (after 28 days of co-culture); incubation with PP2, a broad-spectrum Src kinase inhibitor, completely inhibited host invasion (Wu et al., 2011)

*Infection with *B. garinii*. MOI = Multiplicity of Infection, RB = round body, RT PCR = Real Time Polymerase Chain Reaction.

### Differential Borrelial Attachment and Entry Process Exhibited by Different Cell Lines

Pathogens can highjack cellular actin structures and utilize them for invasion purposes (Rottner et al., 2017; Stradal and Schelhaas, 2018). Several bacteria species are known to exploit cellular surface extensions for attachment and invasion into eukaryotic cells. For example, *Salmonella typhimurium*, a flagellated bacterium, stops and scans the surface of the cell, such as membrane ruffles, for the best entry site (Misselwitz et al., 2012). In a study where primary monocytes were infected with a variety of spirochetes, coiling but not conventional phagocytosis of *B. burgdorferi* could be increased with membrane ruffling inducing chemicals (granulocyte-macrophage colony-stimulating factor and phorbol myristate acetate) (Rittig et al., 1998). Studies with the bacterium *Shigella* have demonstrated a capture mechanism by nanometer thin micropodial extensions, which help the bacteria invade the cell by bringing it into close contact with the cell membrane (Romero et al., 2011). Similarly, invasion of a variety of different host cells by *B. burgdorferi* have been exhibited to include protrusions from the host cell. For example, in phagocytic cells such as macrophages (Hoffmann et al., 2014; Naj and Linder, 2015; Meriläinen et al., 2016) and dendritic cells (Suhonen et al., 2003), as well as in non-phagocytic neuroglial (Williams et al., 2018) and primary synovial cells (Girschick et al., 1996), *B. burgdorferi* was seen internalized via coiling phagocytosis with pseudopod involvement (Table 3). Here *B. burgdorferi* was detected to interact with especially the SW1353 cells, where microscopic, filopodia-like protrusion from the SW1353 cells were observed “grabbing” attached *B. burgdorferi* (Figure 1E). In addition, these interactions facilitated a longitudinal but apical tip entry into the SW1353 cells (Figure 1E), as outlined in previous studies (Szczepanski et al., 1990; Hechemy et al., 1992; Klempner et al., 1993; Klose et al., 2021). Interaction between BJ cells and *B. burgdorferi* was observed less often (Figure 1F) as in other research (Klempner et al., 1993; Rittig et al., 1996), suggesting differences in cellular adhesion by *Borrelia* most likely due to different receptors on the human cells.

*Borrelia* cell invasion has been investigated in several cell lines, such as macrophages (Rittig et al., 1992, 1994; Montgomery and Malawista, 1996; Naj and Linder, 2015; Meriläinen et al., 2016; Klose et al., 2021) and in non-phagocytic cells (Thomas and Comstock, 1989; Georgilis et al., 1992; Klempner et al., 1993; Girschick et al., 1996; Livengood and Gilmore, 2006), as summarized in Table 3. *Borrelia* has been described to be internalized by coiling phagocytosis into macrophages (Rittig et al., 1998; Hoffmann et al., 2014; Naj and Linder, 2015; Meriläinen et al., 2016), human dendritic cells (Filgueira et al., 1996; Rittig et al., 1996; Suhonen et al., 2003), as well as, into neuroglial cells (Livengood and Gilmore, 2006; Williams et al., 2018). Specifically, the protein Daam1 (disheveled-associated activator of morphogenesis) has been identified as a regulator for *Borrelia* uptake by filopodia formation and phagocytosis (Hoffmann et al., 2014; Williams et al., 2018). F-actin rich pseudopodia was demonstrated to be used in the pseudopodia engulfment of *Borrelia* in macrophages (Girschick et al., 1996; Meriläinen et al., 2016). We did not observe such coiling by the host in either cell line, which could be due to the early time point (30 min) of the host cell entry experiment (Figure 1), or because of a completely different invasion mechanism. However, the interaction between SW1353 cells and *Borrelia* described above, suggest some form of actin filament involvement during host invasion in these cells.

Interestingly, all *B. burgdorferi* attached to SW1353 cells at 30 min were spirochetal formed, while the majority of attached *B. burgdorferi* on infected BJ cells were in coiled forms (Figure 1G). *B. burgdorferi* infected murine fibroblast cells exhibited “cyst-like” forms inside these cells (Wu et al., 2011). This suggested alternative entry mechanisms for *Borrelia*, based on the host cell type, as a strategy that may increase immune invasion. Furthermore, Wu et al. (2011) speculated the possibility for the “cyst-like” morphologies to be the result of inactive RB forms, which could revert back to spirochetes after reinstallment into nutritionally replete environment. Moreover, *Borrelia* RBs have been shown to be internalized by coiling phagocytosis less often than spirochetes (Meriläinen et al., 2016), suggesting that the alterations in the membrane receptors affect borrelial invasiveness. Hence, the coiled forms visible in the infected BJ cells observed here (Figure 1F) indicate that there could even be different borrelial membrane interactions required for host cell invasion in BJ than in SW1353 cells.

*Borrelia* infections in HUVEC and HeLa cells have been demonstrated to be time, dose, and temperature dependent (Thomas and Comstock, 1989; Szczepanski et al., 1990; Klempner et al., 1993; Isaacs, 1994). In accordance with previous findings, both infected SW1353 and BJ cells exhibited dose-dependent infectivity by *B. burgdorferi*. However, while the infection increased in a time dependent manner in BJ cells, the amount of infected SW1353 cells peaked at 48 h and decreased subsequently (Figure 2). A similar peak at 48 h has been previously shown with HUVE cells (Ma et al., 1991). Curiously, the HUVE cells used in the abovementioned study were normal, non-immortalized cells, similar to our normal dermal fibroblasts, and yet the time dependency results aligned with the immortalized cancerous SW1353 cell line.

### Intracellular Persistence

Studies have indicated apoptotic cell death induced by *Borrelia* infection in dermal fibroblasts (Rozwadowska et al., 2017), neural cells (Myers et al., 2009; Ramesh et al., 2013), peripheral T lymphocytes (Sandra et al., 2003), and monocytes (Cruz et al., 2008) among others. Contradictory to previous studies, our dermal fibroblast and chondrosarcoma cells grew similarly to the untreated cells despite being infected with a high MOI (200) (Figure 3). However, several other studies have demonstrated corroborating results to ours with viable mammalian cells after long-term borrelial infection (Thomas et al., 1994; Girschick et al., 1996; Livengood and Gilmore, 2006; Wu et al., 2011).

Furthermore, previous studies have revealed that *B. burgdorferi* can be regrown after being internalized into human cells (Georgilis et al., 1992; Thomas et al., 1994; Livengood and Gilmore, 2006; Wu et al., 2011). Moreover, by analyzing borrelial gene expression with real-time PCR, *Borrelia* have been shown to be metabolically active while internalized inside mouse fibroblast cells (Wu et al., 2011). We also observed *B. burgdorferi* regrowth from washed and pelleted 24 and 72 h infection co-cultures in BSK II supernatant. Although numerous motile spirochetes were observed in only a few samples from both cell lines during the 6-week time period (Figure 4), these results supported the previous studies mentioned above, where *B. burgdorferi* regrowth after internalization into human cells was achieved. Hence, similar to previous hypotheses, we propose that both the lack of cytopathic effects and the ability to regrow *Borrelia* from co-cultured samples after removal of external bacteria, suggested that these non-phagocytic cells could serve as a hiding site for *Borrelia* to avoid the host immune system, while simultaneously inducing sustained infection in the host (Ma et al., 1991; Klempner et al., 1993; Girschick et al., 1996; Embers et al., 2004; Livengood and Gilmore, 2006; Wu et al., 2011; Naj and Linder, 2015).

By using cryo-EM, borrelial intracellular location in non-phagocytic human cells could be investigated at an ultrastructural level. Even after 9 days post-infection *B. burgdorferi* was visualized inside the infected human cells at a variety of locations (Figure 5). Previous studies have reported *B. burgdorferi* freely in the cytosol, and without signs of degradation, of primary synovial cells after 5 d of co-culture (Girschick et al., 1996). Similarly, in HUVE cells, *B. burgdorferi* was identified moving through the cell monolayer most likely through the tight junctions (Szczepanski et al., 1990), or through the cytoplasm of the host cells (Comstock and Thomas, 1991). Furthermore, in EM visualized infected HUVEC samples, intracellular *B. burgdorferi* was seen surrounded by host membrane (Comstock and Thomas, 1989). Investigation of *B. burgdorferi* infected Vero cells demonstrated the problematic nature of borrelial intracellularity, since the researchers were unable to determine whether *Borrelia* was freely in the cell cytoplasm or enclosed by a membrane (Hechemy et al., 1992). Here, we noticed the bacteria inside these cells both freely in the cytoplasm and enclosed within the host membrane (Figure 5). Since the human cells survived, even flourished, during long-term infection with *B. burgdorferi* (Figure 3), it was speculated that *B. burgdorferi* utilized the variety of locations inside these cells in order to first escape the possibly hostile external environment, and later being processed by the endosomal processing pathways.

*Borrelia* have been shown to be processed in the lysosomes of macrophages (Rittig et al., 1994; Montgomery and Malawista, 1996; Naj and Linder, 2015; Meriläinen et al., 2016) and phagolysosomes of dendritic cells (Suhonen et al., 2003). Specifically, we have previously demonstrated that at 24 h, spirochetes co-localized with macrophage lysosome-associated membrane protein 2 (Lamp2) significantly more than RBs (Meriläinen et al., 2016). Here, however, in a co-localization experiment with *Borrelia* and Lamp2, we witnessed a negligible amount of co-localization between *B. burgdorferi* and lysosomes (Figure 6 and Table 2). Similar to previous findings in macrophage (Klose et al., 2019), *B. burgdorferi* was observed in coiled, rounded, and at times damaged-looking forms in SW1353 cells (Figure 6 and Table 2). Additionally, *Borrelia* was more co-localized with lysosomes at day 9 post-infection (Table 2), suggesting that *B. burgdorferi* is more actively processed in SW1353 than in BJ cells.

On the other hand, borrelial degradation without lysosomal activity has been demonstrated as well in macrophages and dendritic cells (Rittig et al., 1996). Our previous findings with macrophages have demonstrated that since RBs co-localized with lysosomes less than spirochetes, it would indicate an alternative processing route for RBs in macrophages (Meriläinen et al., 2016). Here, similarly to Meriläinen et al. (2016), since *B. burgdorferi* was observed in other forms than spirochetes in the SW1353 cells (Figures 6A,B) it would suggest an alternate processing pathway in these cells.

Furthermore, *Borrelia* has been found as elongated spirochetes in the cytoplasm of macrophages (Naj and Linder, 2015), dendritic cells (Filgueira et al., 1996; Suhonen et al., 2003), fibroblasts (Klempner et al., 1993), HUVECs (Thomas and Comstock, 1989; Szczepanski et al., 1990), and synovial cells (Girschick et al., 1996). Similarly, here *B. burgdorferi* was seen in the cytoplasm of the human cells. Specifically, as exhibited with infected BJ cells (Figures 2D, 6C,D), *Borrelia* did not look damaged and the spirochete appeared free in the cytoplasm, corresponding with the above-mentioned previous results. Again, we demonstrated that *B. burgdorferi* was handled differently between the two human cell lines and indeed can evade the lysosomal pathway aiding in its persistence.

### Mechanism of Persistence by Pleomorphic Forms

In order for *Borrelia* to persist in the infected host, it must escape the host immune system. One possible mechanism for borrelial immune evasion currently gaining more support is the persister forms of *Borrelia* (Rudenko et al., 2019). Being a pleomorphic bacterium, *Borrelia* can alter its shape to a metabolically inactive round body, and establish a microcolony or biofilm, especially in unfavorable culture conditions. Specifically, RBs can be induced by osmotic pressure already in 10 min, but also with serum starvation, high temperatures, and changes in pH (Alban et al., 2000; Murgia and Cinco, 2004; Meriläinen et al., 2015; Sharma et al., 2015). Moreover, the formation of biofilms was observed to be temperature, pH, and growth phase dependent (Srivastava and De Silva, 2009). The current investigation demonstrated a marked difference in *B. burgdorferi* pleomorphic forms during a total of 9 days infection period between infected human fibroblast and chondrosarcoma cells. While the majority of *B. burgdorferi* remained as spirochetes during BJ infections, in infected SW1353 cells mostly blebs and RBs, as well as, damaged forms of *B. burgdorferi* were observed (Figures 7A,B). “Cyst-like,” globular forms of *Borrelia* have been recorded from infected murine fibroblasts (Wu et al., 2011), in HUVE and neuroglial cells (Livengood and Gilmore, 2006), as well as, in macrophages (Naj and Linder, 2015). Naj and Linder (2015) suggested that borrelial compaction into globular forms is in response to the elongated shape of the spirochete, although they also observed a loss of membrane in the phagosomal surface. Since the cell lines used here were non-phagocytic, the observed pleomorphic forms were thought to occur due to the bacterial response to its environment rather than the host cell response to the bacterium.

Studies have noticed that the reversion of long-term H_2_O induced RBs took at least 3 months to revert back to spirochetes (Brorson and Brorson, 1998; Gruntar et al., 2001; Murgia and Cinco, 2004). Hence, the attempts to cultivate *Borrelia* from patient samples should take this into account and survey the samples for pleomorphic forms, as well. As can be seen from Figure 7A, synovial tissue samples could contain other borrelial pleomorphic forms than spirochetes. Furthermore, the damaged *Borrelia* could cause a prolonged immune reaction, resulting in chronic inflammation of the joints without the existence of live bacteria (Carlson et al., 1999).

## Conclusion

In this study, we demonstrated how *B. burgdorferi* exploited different mechanisms in two human cell lines, non-immune and non-phagocytic, to aid in its persistence. The utilization of the host cell surface extensions and differences in borrelial shapes while invading the host cells, as well as the differences in intracellular handling of the bacteria, confer *Borrelia* fitness for survival. Intracellular persistence of *Borrelia*, due to avoidance of lysosomal co-localization, lack of cytopathic effects, and the ability to change its shape, all provide strategies *Borrelia* can employ for immune evasion and persistence.

## Data Availability Statement

The original contributions presented in the study are included in the article/Supplementary Material, further inquiries can be directed to the corresponding author/s.

## Author Contributions

KK took part in conceptualizing the study, performed all the experiments, analyzed the data, and wrote the original draft. JN provided assistance in the lab, in the data analysis, and reviewed the draft. VM supervised the EM, co-localization, and GFP experiments, and reviewed the draft. LG conceptualized the overall study, participated in data analysis, and reviewed the draft. All authors contributed to the article and approved the submitted version.

## Conflict of Interest

Author LG is employed by Te?ted Oy, Jyväskylä, Finland. The remaining authors declare that the research was conducted in the absence of any commercial or financial relationships that could be construed as a potential conflict of interest.

## Publisher’s Note

All claims expressed in this article are solely those of the authors and do not necessarily represent those of their affiliated organizations, or those of the publisher, the editors and the reviewers. Any product that may be evaluated in this article, or claim that may be made by its manufacturer, is not guaranteed or endorsed by the publisher.
