# Supplementary material for: Distinctive Evasion Mechanisms to Allow Persistence of Borrelia burgdorferi in Different Human Cell Lines
Source: Front Microbiol. 2021 Oct 12;12:711291. doi: 10.3389/fmicb.2021.711291 (PMC8546339; doi:10.3389/fmicb.2021.711291)
Supplement: Supplementary file 1 [file Data_Sheet_1.docx]

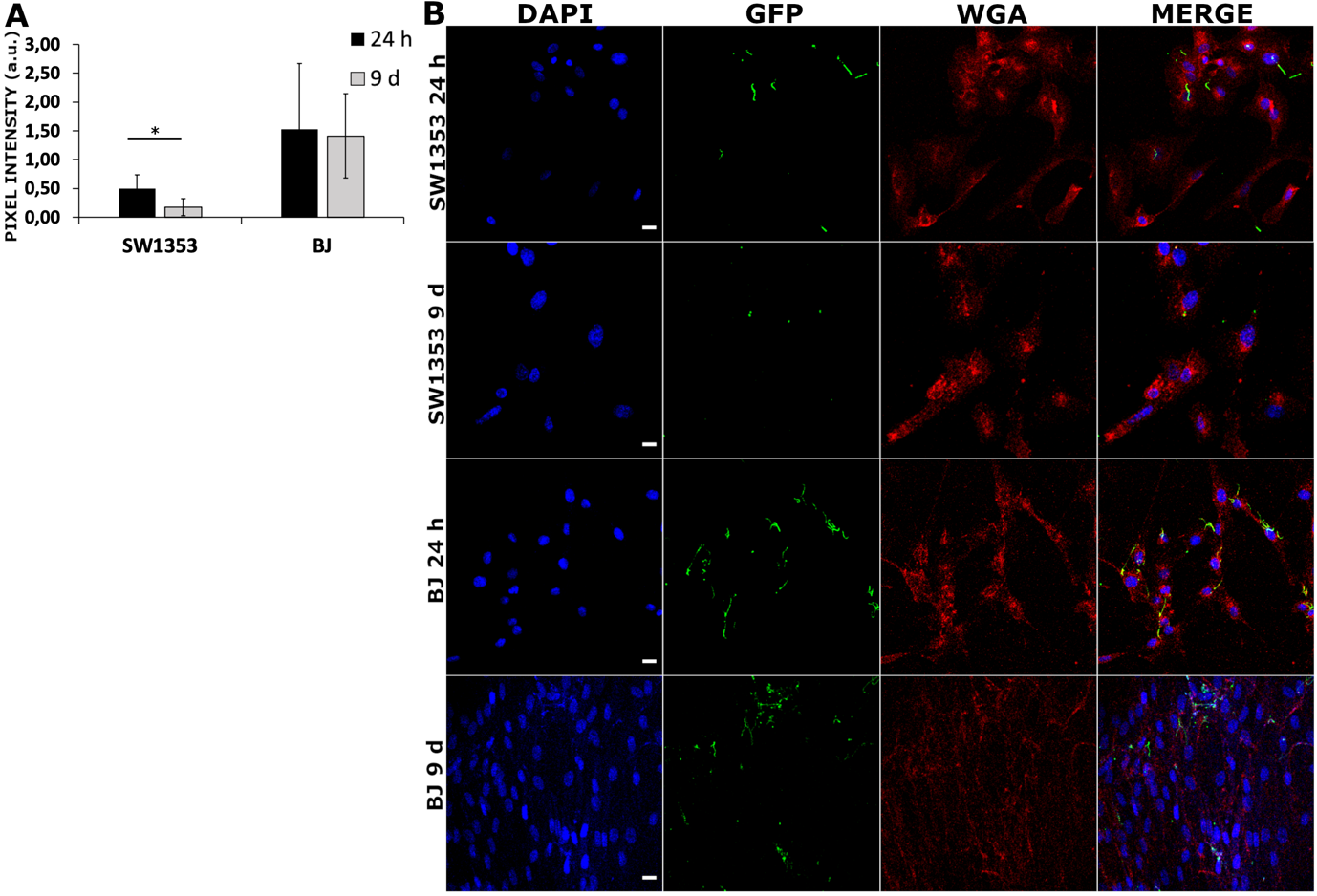


**Figure S1. *B. burgdorferi* infection varied between cell lines.** A GFP signal measurement was performed between 24 h and 9 d samples of SW1353 and BJ cells infected with *B. burgdorferi* (MOI 40). (A) GFP signal did not diminish significantly in BJ cells, while there was a clear drop in the SW1353 cell line (*p-value* ≤ 0.005). (B) Representative images of infected SW1353 cells from 24 h (top row) and 9 d (second row) post infection, and BJ cells from the same time points (24 h in third row, 9 d last row). The cell nucleus (DAPI) fluorescence blue, *B. burgdorferi* (GFP) green, and the cell membrane (WGA) as red. Merged images are provided. Scale bars 10 μm.


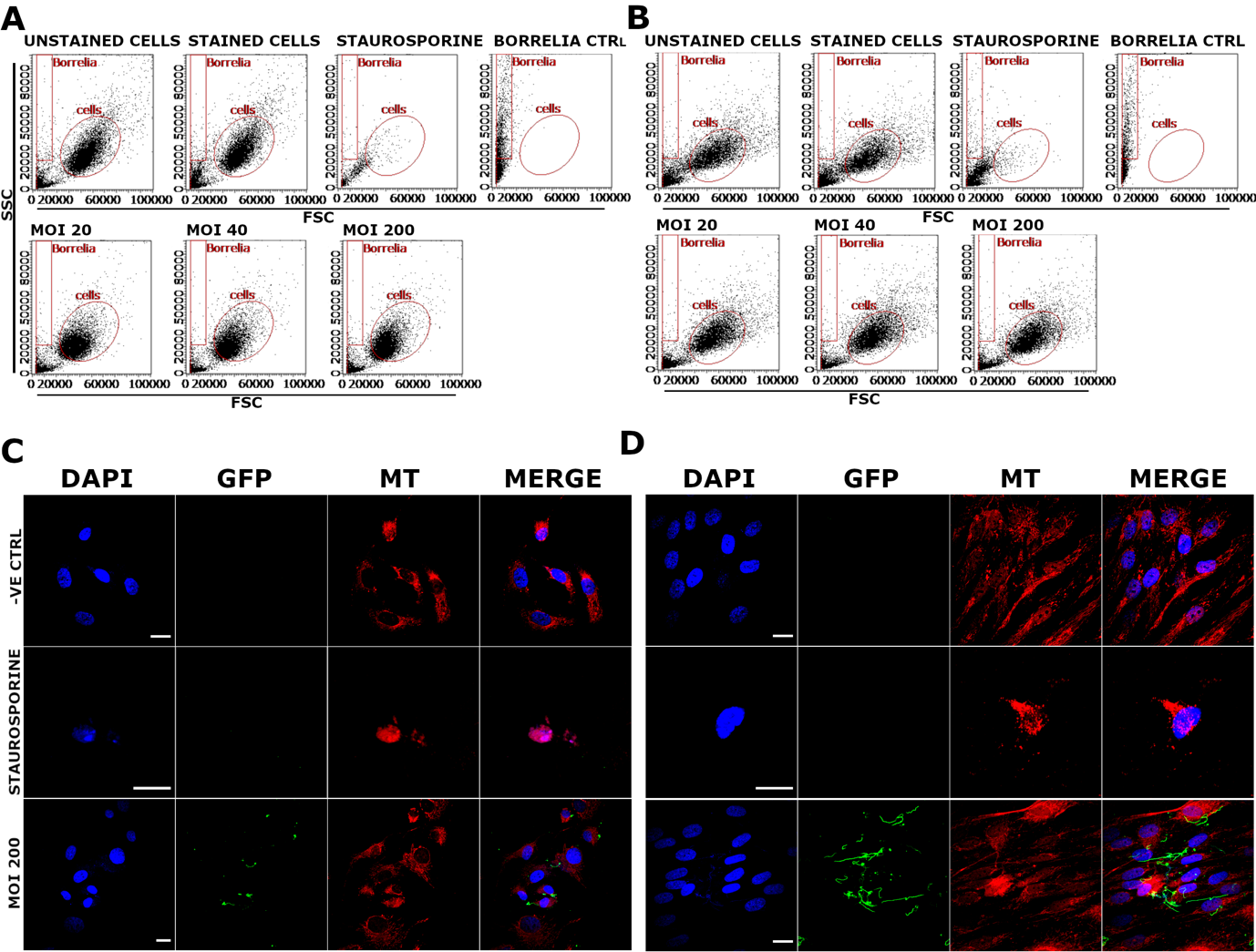


**Figure S2. Viability of the human cells after *B. burgdorferi* infection was further illustrated by staining for live mitochondria.** (A) and (B), representative dot blots of 96 h of unstained, stained, and staurosporine treated SW1353 (A) and BJ (B) cells, with Borrelia only controls and the infected samples (MOIs 20, 40 and 200). Side scatter (SSC) on the y-axis and forward scatter (FSC) on the x-axis. Cell populations are red circles in the middle, while Borrelia are red squares on the left.

Confocal microscope images of MitoTracker™ stained *B. burgdorferi* infected SW1353 (C) and BJ (D) cells at 96 h. The cell nucleus (DAPI) fluorescence blue, *B. burgdorferi* (GFP) green, and the mitochondria (MT) as red. Merged images are provided to display overlay of the channels. Scale bars 20 μm.
